# From pixels to prediction: improving imaging through AI in rheumatology

**DOI:** 10.1016/j.ero.2026.100203

**Published:** 2026-06-20

**Authors:** Peter Mandl, Xenofon Baraliakos, Vincent Bouget, Peter Böhm, Martin Brom, Michael Deman, Thomas Hügle, Arnd Kleyer, Georg Langs, Anastasiia Miendrova, Isabel Morales Ivorra, Joeri Nicolaes, Anders Nordell, Mikkel Østergaard, Costantino Pitzalis, Karin Rainer, Kristian Reich, Thiusius Rajeeth Savarimuthu, Daniela Sieghart, Clemens Watzenböck, Loreto Carmona, Robert Janiczek, Daniel Aletaha

**Affiliations:** 1 Division of Rheumatology, Medical University of Vienna, Vienna, Austria; 2 Ludwig Boltzmann Institute of Arthritis and Rehabilitation, Vienna, Austria; 3 Rheumazentrum Ruhrgebiet Herne, Ruhr-University Bochum, Herne, Germany; 4 Scienta Lab, Paris, France; 5 Patient Research Partner, EULAR, Kilchberg, Switzerland; 6 Translational Medicine, Novartis Biomedical Research, Novartis Campus, Basel, Switzerland; 7 Janssen Pharmaceutica NV, a Johnson & Johnson Company, Beerse, Belgium; 8 Department of Rheumatology, University Hospital Lausanne (CHUV) and University of Lausanne, Lausanne, Switzerland; 9 Department of Rheumatology and Clinical Immunology, Charité-Universitätsmedizin Berlin, Campus Charité Mitte, Berlin, Germany; 10 Department of Biomedical Imaging and Image-guided Therapy, Medical University of Vienna, Vienna, Austria; 11 Comprehensive Center for Artificial Intelligence in Medicine, Medical University of Vienna, Vienna, Austria; 12 European Alliance of Associations for Rheumatology (EULAR), Kilchberg, Switzerland; 13 Singularity Biomed, Sant Cugat del Vallès, Spain; 14 UCB Pharma, Brussels, Belgium; 15 Department of Electrical Engineering (ESAT), Center for Processing Speech and Images, KU Leuven, Leuven, Belgium; 16 Collective Minds Radiology, Danderyd, Sweden; 17 Copenhagen Center for Arthritis Research, Center for Rheumatology and Spine Diseases, Rigshospitalet, Glostrup, Denmark; 18 Department of Clinical Medicine, Faculty of Health and Medical Sciences, University of Copenhagen, Copenhagen, Denmark; 19 Centre for Experimental Medicine & Rheumatology, William Harvey Research Institute, Faculty of Medicine and Dentistry, Queen Mary University of London, London, UK; 20 Barts Health NHS Trust and National Institute for Health and Care Research (NIHR) Barts Biomedical Research Centre (BRC), Charterhouse Square, London, London, UK; 21 Austrian Agency for Health and Food Safety GmbH, Vienna, Austria; 22 MoonLake Immunotherapeutics AG, Zug, Switzerland; 23 Translational Research in Inflammatory Skin Diseases, Institute for Health Services Research in Dermatology and Nursing, University Medical Center Hamburg-Eppendorf, Hamburg, Germany; 24 ROPCA ApS, Odense, Denmark; 25 University of Southern Denmark, Odense, Denmark; 26 Instituto de Salud Musculoesquelética, Madrid, Spain; 27 Johnson & Johnson, Spring House, PA, USA

## Abstract

Imaging plays a central role in the diagnosis, monitoring, and prediction of rheumatic and musculoskeletal diseases (RMDs), yet its clinical and regulatory impact remains limited by variability, the lack of standardisation and metrics for quantification, and slow clinical translation. Artificial intelligence (AI) offers unique opportunities to overcome these barriers through automated segmentation, quantitative feature extraction, and multimodal data integration. The AutoPiX consortium seeks to design and validate machine-learning–driven imaging pipelines to expedite the translation of imaging data into quantitative biomarkers, facilitate their systematic characterisation, and promote their use in enhancing patient stratification and bringing imaging-based endpoints closer to regulatory endorsement in clinical trials. This paper outlines unmet needs, the potential translational impact of AI in RMDs and the perspectives of key stakeholders, including clinicians, patients, regulators, and industry partners. We call for harmonised imaging protocols, large-scale imaging datasets, cross-disciplinary collaboration, and early regulatory engagement to realise the transformative potential of AI-enhanced imaging biomarkers.

## IMAGING AS A BIOMARKER

Imaging modalities, such as radiography, ultrasound, and magnetic resonance imaging (MRI), have long been essential diagnostic and monitoring tools in rheumatic and musculoskeletal diseases (RMDs), especially in inflammatory arthritis, enabling clinicians to detect or monitor inflammation and tissue damage in ways that clinical and serological markers can hardly capture. Large numbers of medical images are acquired along the typical patient journey in RMDs, and imaging is an integral part of most clinical trials. In current clinical practice, the information enclosed in images is restricted to narrative reviews composed by radiologists, which, while helpful in clinical practice, are both incomprehensible to patients and have only limited utility for monitoring changes.

To leverage this information better, images need to be quantified, that is, they first need to be transformed into biomarkers—scoring systems developed to grade/rate a particular element or feature of the image. Over the past decades, numerous imaging biomarkers, such as scoring systems used to assess radiological progression on radiographs [1,2] have been developed and validated, thus becoming key outcomes for structural damage label claims. Imaging provides an objective and quantifiable measure of disease-modifying capacity, demonstrating a drug’s ability to slow or prevent irreversible joint destruction, which is a primary goal for regulators like the European Medicines Agency [3] and the U.S. Food and Drug Administration [4]. Unlike radiographs, ultrasound and MRI can also depict signs of inflammation as well as of structural damage. Scoring systems for these and other modalities have been developed to quantify such changes in peripheral and axial joints, and have emerged as key outcomes in clinical trials [[5], [6], [7], [8], [9], [10]].

Despite incremental improvements, imaging biomarkers remain underutilised in clinical decision-making and regulatory contexts. They are often semiquantitative and depend on expert scoring, introducing intra- and inter-reader variability, which remain major limitations [11]. Acquisition, scoring, and reporting protocols may differ widely, hampering standardisation across centres. Translation into clinical care has been slow, partly due to resource constraints and limited access to trained personnel and advanced equipment. Although scoring systems are approved endpoints in clinical trials in peripheral (radiography) and axial (radiography, MRI) forms of arthritis, this is not the case for MRI or ultrasound of the peripheral joints, hampering their ability to accelerate therapeutic development.

This landscape highlights critical unmet needs: automation, reproducibility, and validation to demonstrate added predictive and prognostic value. Without addressing these limitations, the full potential of imaging biomarkers in rheumatology will remain unrealised.

## POTENTIAL AND OPPORTUNITIES FOR AI IN IMAGING

Recent advances in artificial intelligence (AI) in musculoskeletal imaging hold the promise to overcome many of the above-mentioned shortcomings. Deep learning (DL), in particular convolutional neural networks (CNN) and vision transformer (ViT) architectures, enables hierarchical representation learning directly from raw imaging data. Although automated segmentation algorithms can robustly delineate structures such as cartilage, synovium, and bone, detecting and classifying disease with high reproducibility, contemporary DL approaches increasingly rely on end-to-end architectures that learn hierarchical and task-specific feature representation directly from the data. CNNs, ViTs, and hybrid models can capture subtle spatial, textural or temporal patterns that are invisible to human visual perception or conventional scoring without predefined feature engineering, enabling automated disease detection, grading, and prognostication with high reproducibility and reduced operator dependency [[12], [13], [14]]. This offers opportunities for earlier diagnosis and intervention and for a more fine-grained continuous assessment of disease progression or treatment response in patients.

Predictive models trained on large, multimodal datasets can scale up clinical validation and improve the selection and stratification of patients according to prognosis or therapeutic response, which would have implications for both routine practice and clinical trials. Of note, the field is rapidly moving towards self-supervised and foundation-model (FM) paradigms, whereby models are pretrained on large volumes of unlabelled imaging data to learn generalisable representations before they are fine-tuned to achieve specific tasks. This reduces dependence on expert annotations, a major bottleneck in any form of imaging, and enhances cross-centre robustness. Such approaches are particularly relevant and timely in multicentre settings, where system variability and protocol heterogeneity have historically limited harmonisation. Domain adaptation and federated learning frameworks further enable collaborative model training without centralised data sharing, addressing regulatory and privacy constraints while preserving scalability.

An additional advantage of self-supervised learning and FM lies in their enhanced performance. By leveraging large-scale, partially annotated or unlabelled datasets to learn transferable imaging representations, they can improve generalisability across centres and imaging protocols. Importantly, the reproducibility and scalability of AI-based analyses enable intercentre alignment, a long-standing barrier to multicentre clinical trials [15]. Beyond single-task prediction, AI systems are increasingly becoming capable of multimodal and longitudinal modelling. Joint learning frameworks have the capacity to integrate imaging with clinical parameters, laboratory values, genomics, proteomics, and electronic health records to generate patient-specific risk signatures rather than utilising isolated imaging biomarkers. Temporal models incorporating serial imaging data allow continuous disease trajectory modelling, potentially redefining how progression and treatment response are quantified.

Imaging biomarkers, when embedded within multidimensional and longitudinal patient models, can contribute to refined phenotyping, improved stratification in clinical trials, and more adaptive therapeutic decision-making [16]. Rather than static classification, these approaches support dynamic, individualised prediction of disease emergence, flare risk, structural damage development, or therapeutic response.

Generative AI methods also offer emerging opportunities. Synthetic data generation and image-to-image translation can support data augmentation, protocol harmonisation, and simulation of disease progression states. In parallel, explainable AI techniques are improving interpretability by highlighting salient regions or latent features associated with predictions, thereby facilitating clinical trust and regulatory acceptance. Collectively, these developments position AI not merely as an automation tool but as a scalable infrastructure for precision medicine.

## TRANSLATIONAL AND CLINICAL IMPACT OF AutoPiX

AutoPiX is an Innovative Health Initiative (IHI)-funded consortium that brings together pharmaceutical and medical technology partners with leading academic institutions, patients, and regulators to maximise the utility of imaging biomarkers to the benefit of patients with arthritis, supporting more precise diagnosis, improved monitoring, and personalised treatment approaches. The main goals of AutoPiX are shown in the Table.

**Table:** Main goals of AutoPiX

Goal	Description
Creation of a data lake	Building a large-scale imaging data lake to nurture future European research on AI for imaging in rheumatology
Imaging biomarker quantification	Validating existing and developing new imaging-based as well as multimodal ML algorithms and foundation models using the largest collection of images of patients with arthritis from both academia and industry partners
Imaging biomarker utilisation	Advance novel imaging techniques, such as self-performed imaging (eg, smartphone-acquired photograph- and single-camera motion capture video-based detection tools) [17,18], thermography [19], robotic-assisted ultrasound [20], or novel nuclear imaging applications [21,22].
Interoperability and trust	Develop transparent and trustworthy AI pipelines that can be used across centres. Harmonised and standardised approaches will facilitate regulatory engagement and eventual acceptance of imaging biomarkers as surrogate endpoints.
Impact the design of clinical trials	More sensitive measures of structural damage progression as well as patient stratification based on AI-derived imaging features may enable enrichment of trial populations. Improving the likelihood of detecting treatment effects, shortening trial duration and cost, and thereby expediting drug development.
Creation of a visual foundation model (FM)	Develop the first multimodal foundation model dedicated to rheumatology by integrating conventional radiography (CR), ultrasound (US), and MRI data. The model will employ contrastive and semisupervised learning to enhance existing diagnostic and prognostic AI algorithms, serving as the technological foundation for a research and clinical support ecosystem in rheumatology.
Impact routine clinical care	Support personalised treatment selection, longitudinal monitoring, and early identification of disease flares or progression.

AI, artificial intelligence; ML, machine learning; MRI, magnetic resonance imaging.

Ultimately, AutoPiX aims to have an impact on several touchpoints of the patient journey, leveraging existing as well as novel technologies and international collaboration. Its work packages align with different phases of the patient journey, as illustrated in the Figure.

**Figure:**
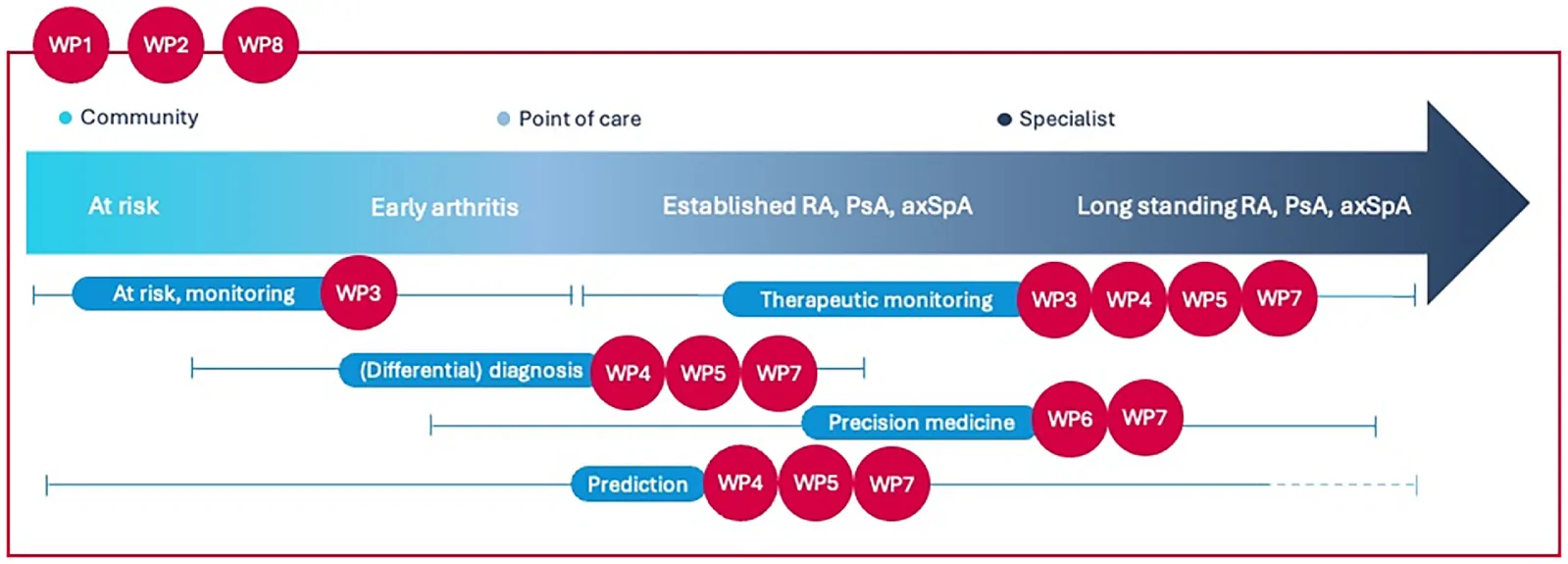
Touchpoints of the patient journey that will be impacted by the results of the AutoPiX project and the respective work packages (WP). WP1 is responsible for project management and governance; WP2 is responsible for the setup of the Data Lake; WP3 focuses on self-monitoring and point-of-care imaging; WP4 and WP5 are dedicated to building the AI models for peripheral and axial arthritis respectively; WP6 aims at the biologic validation of imaging biomarkers by investigating the correlation of imaging and histopathologic findings; WP7 is dedicated to the interactions with the health ecosystem - from health care professionals and hospitals to regulatory authorities, and WP8 is in charge of the dissemination, exploitation and sustainability of the outcomes of AutoPix. axSpA, axial spondyloarthritis; PSA, psoriatic arthritis; RA, rheumatoid arthritis.

## STAKEHOLDER PERSPECTIVES AND NEEDS

The successful integration of AI-enhanced imaging biomarkers in rheumatology requires the active involvement and alignment of multiple stakeholders, each with distinct priorities, needs, and concerns that must be acknowledged and addressed to ensure adoption, trust, and long-term sustainability.

## PATIENTS

Patients with RMDs are increasingly recognised as vital partners in research and care, consistent with the EULAR framework for involving patient research partners in rheumatology research [23]. Patients require AI pipelines that are transparent, trustworthy, safe, and which may ultimately convey imaging results in lay language for clear understanding [24,25]. Patient concerns about the potential of AI for misdiagnosis, privacy breaches, and reduced time with clinicians need to be acknowledged and addressed, and patients should be included in the conversation about integrating AI into clinical care and decision-making processes. Targeted educational initiatives may improve patient’s understanding of and engagement with AI-driven healthcare.

## CLINICIANS

For clinicians, imaging is both a diagnostic and a monitoring tool, the use of which, however, is hampered by variability in image acquisition (eg, ultrasound), interpretation (eg, MRI), time-consuming scoring, accessibility (eg, positron emission tomography), and the lack of universally accepted quantitative metrics. Elevating imaging to the next level demands robust, interpretable, and seamlessly integrated ML algorithms within healthcare workflows, capable of harnessing the incredible amount of information captured in images, most of which has not yet been exploited. Automated systems would enable physicians not versed in the systematic scoring of radiographs and other techniques to gauge their sensitivity and, in particular, to detect clinically relevant changes earlier. Ultimately, this would also reduce the routine workload for radiologists, who could then invest their expertise in complex cases and other time-consuming differential diagnoses.

The translation of AI into routine care, however, requires rigorous external and prospective validation, harmonisation across imaging platforms, and regulatory approval as detailed below. Particular attention must be paid to bias, explainability, and the potential risks of under- or overdiagnosis. AI systems should therefore be implemented as decision-support tools under clinical supervision rather than as autonomous diagnostic systems.

## REGULATORS

Regulatory agencies play a pivotal role in determining the acceptance of imaging biomarkers and the approval of AI tools in healthcare [26,27]. They have already accepted imaging biomarkers in RMDs, and this precedent highlights the path towards regulatory acceptance for others to come when supported by strong validation data. A similar pathway could be envisaged for AI-enhanced imaging biomarkers in RMDs, provided that initiatives such as AutoPiX can deliver harmonised datasets, reproducibility evidence, and clinical validation.

## PHARMACEUTICAL AND TECHNOLOGICAL INDUSTRY

Pharmaceutical companies, medical imaging vendors, and AI technology providers are essential drivers of innovation with differing roles and needs. In oncology, partnerships between pharma companies and imaging AI vendors have enabled the qualification of radiomic biomarkers as exploratory endpoints in drug trials [28]. A comparable model in rheumatology—where pharma partners leverage AutoPiX pipelines to integrate imaging biomarkers into early-phase drug development and clinical trial design—could accelerate adoption and establish a precedent for broader use.

## CHALLENGES AND ETHICAL CONSIDERATIONS

AI is not a universal cure. Algorithmic bias is an important point of attention: outcomes can only be as good as the datasets used for training, whereas models trained on nonrepresentative data risk embedding disparities in healthcare delivery.

Lack of external validation across diverse cohorts limits generalisability, whereas opaque ‘black-box’ models hinder clinician trust [29]. Addressing governance challenges, including secure data sharing and compliance with data protection regulations such as General Data Protection Regulation [30], takes time. Risk-based approaches, including data protection impact assessments, can help identify and manage privacy risks to individuals based on the likelihood and impact of potential harm resulting from the processing of their health data.

Beyond technical performance, the use of AI for image interpretation raises specific ethical and legal questions, including accountability for diagnostic errors, transparency of automated decision-making, and the delineation of responsibility between clinicians, institutions, and technology developers. Clear regulatory frameworks and medico-legal guidance are essential to ensure that AI-supported decisions remain subject to appropriate human oversight and professional standards.

Interdisciplinary collaboration between clinical, imaging-, and AI experts underpins the proposed approaches, ensuring that state-of-the-art methodologies are applied appropriately.

Successful integration requires clinician training, workflow adaptation, and transparent reporting of model outputs. Tools must be designed to augment, not replace, clinician expertise.

Developing initial AI models is only the beginning; extensive validation of pretrained models and assembling appropriate datasets, processes, and regulatory documentation require significant additional effort. Ensuring generalisability and mitigating bias remain essential steps towards fairness, equity, and clinical trustworthiness. Without these safeguards, AI systems risk worsening disparities rather than reducing them.

## POSITION STATEMENT AND CALL TO ACTION

We contend that imaging biomarkers, enhanced by AI, must be prioritised as essential tools in precision medicine for RMDs.

We call for:

• **Sustainable large-scale imaging datasets** with linked metadata derived from both academia and industry to build the largest imaging data repository in RMDs to nurture future EU R&D programs and enable validation and development of ML models.
• **Harmonised imaging protocols** across centres to enable reproducible and comparable datasets.
• **Training initiatives** in AI and imaging, such as dedicated webinar series, to raise awareness, build capacity, and ensure critical appraisal skills among clinicians, researchers, as well as patients.
• **Cross-disciplinary collaboration** between academia, industry, regulators, and patient representatives to ensure alignment and shared responsibility.
• **Early regulatory engagement** to fast-track biomarker qualification and establish guidelines for clinical adoption.

## CONCLUSION

Imaging biomarkers, when combined with AI, hold transformative potential for rheumatology. They may accelerate drug development, support personalised care, and reduce healthcare variability. AutoPiX provides the infrastructure and momentum to realise this vision. However, success requires coordinated efforts in training, governance, validation, and ethics. Now is the time to unify efforts to ensure that imaging biomarkers reach their full potential and deliver tangible benefits for patients living with RMDs.

## Editor disclosure

The peer review process did not involve Editorial Board Members Daniel Aletaha, Xenofon Baraliakos, Arnd Kleyer, and Mikkel Østergaard, and the editorial decision-making was led by editors who were not involved in the creation of this manuscript.

## Competing interests

PM has received research grants from AbbVie, Alfasigma, Novartis, BMS, Eli Lilly, Janssen, Sobi, and UCB and speaker fees from AbbVie and Sobi. VB holds stocks of Scienta Lab. MB is an employee of Novartis and holds stocks of Novartis and Sandoz. JN is an employee and potential shareholder of UCB Pharma. MØ has received research grants from AbbVie, Amgen, BMS, Merck, Celgene, Eli Lilly, Novartis, and UCB; speaker fees from AbbVie, BMS, Celgene, Eli-Lilly, Galapagos, Gilead, Janssen, MEDAC, Merck, Novartis, Pfizer, Sandoz, and UCB; and consultancy fees from AbbVie, BMS, Celgene, Eli-Lilly, Galapagos, Gilead, Janssen, Merck, Novartis, Pfizer, and UCB. GL has received research grants from NVIDIA and Siemens Healthineers; speaker fees from Roche; and is co-founder and shareholder of contextflow GmbH and NVIDIA. IMI is a shareholder of Singularity Biomed SL. RJ and MD are employees and potential shareholders of Johnson & Johnson. TRS is a co-founder of ROPCA Aps. RJ is a potential shareholder of GSK. TH is a potential shareholder of Atreon SA and has received speaker fees from AbbVie, GSK, Eli Lilly, Johnson & Johnson, UCB, and Sandoz. DA has received consultancy fees from Advanz, AstraZeneca, Janssen, Mitsubishi, Sanofi, and UCB and speaker fees from Advanz, AstraZeneca, Janssen, Mitsubishi, Sanofi, and UCB. XB has received research grants from Novartis, AbbVie, Celltrion, Janssen, and Moonlake; consulting fees from AbbVie, Advanz, Alexion, Alphasigma, Amgen, BMS, Cesas, Celltrion, Clarivate, Galapagos, Greywolfe, J&J, Lilly, Moonlake, Novartis, Peervoice, Pfizer, Roche, Sandoz, Springer, Stada, Takeda, UCB, and Zuellig; speaker fees from AbbVie, Advanz, Alexion, Alphasigma, Amgen, BMS, Cesas, Celltrion, Clarivate, Galapagos, Greywolfe, J&J, Lilly, Moonlake, Novartis, Peervoice, Pfizer, Roche, Sandoz, Springer, Stada, Takeda, UCB, and Zuellig; and Participation on a Data Safety Monitoring Board or Advisory Board from AbbVie, Advanz, Alexion, Alphasigma, Amgen, BMS, Cesas, Celltrion, Clarivate, Galapagos, Greywolfe, J&J, Lilly, Moonlake, Novartis, Peervoice, Pfizer, Roche, Sandoz, Springer, Stada, Takeda, UCB, and Zuellig. He is EULAR President, ASAS Past President, and Editorial Board Member of *Annals of Rheumatic Diseases*.
